# Supplementary material for: Grain Characteristics, Moisture, and Specific Peptides Produced by Ustilaginoidea virens Contribute to False Smut Disease in Rice (Oryza sativa L.)
Source: Biomolecules. 2023 Apr 12;13(4):669. doi: 10.3390/biom13040669 (PMC10136112; doi:10.3390/biom13040669)
Supplement: Supplementary file 1 [file biomolecules-13-00669-s001.zip › biomolecules-2183569-supplementary.pdf]

Supplementary Data

Grain characteristics, moisture, and specific peptides produced by *Ustilaginoidea virens* contribute to false–smut disease in rice (*Oryza sativa* L.)

Robinson C. Jose[1,2], Thangjam Kanchal[2], Bengyella Louis[3], Narayan C. Talukdar[1,4], Devasish Chowdhury[1*]

[1]Institute of Advanced Study in Science and Technology, Guwahati, 781035, Assam, India.

[2]Institute of Bioresources and Sustainable Development (IBSD), Takyelpat, Imphal – 795001, Manipur, India.

[3]Department of Plant Sciences, 19 Tyson Building, University Park, Pennsylvania State University, 16802, PA, USA

[4]Assam down town University Sankar Madhab Path, Gandhi Nagar, Panikahiti, Guwahati-781026, Assam, India

Correspondence email: devasish@iasst.gov.in, Devasish Chowdhury

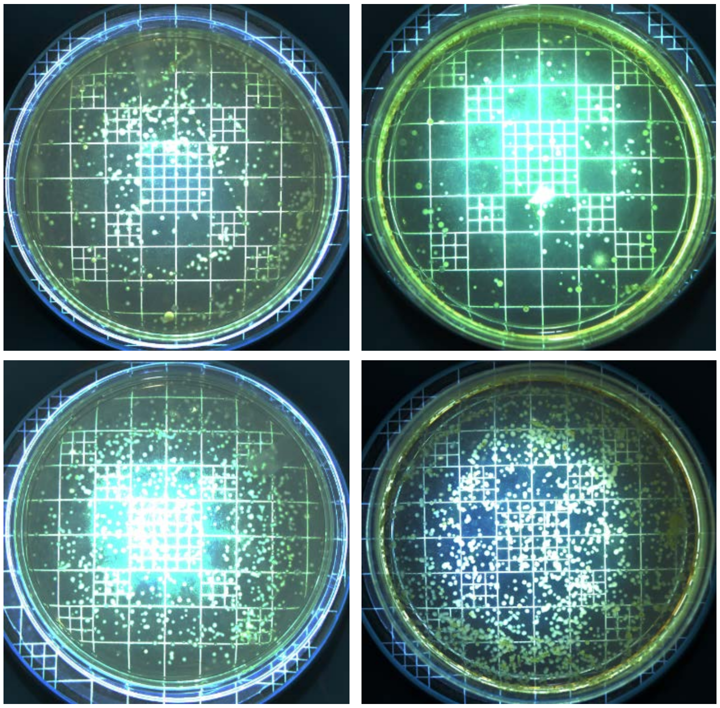

Fig. S1. Colony morphology of *U. virens* at 36 h post-germination at 25° C. The pathogen was isolated from rice smut balls and plated on PDA media containing the antibiotics chloramphenicol (25 mg L^{-1}).

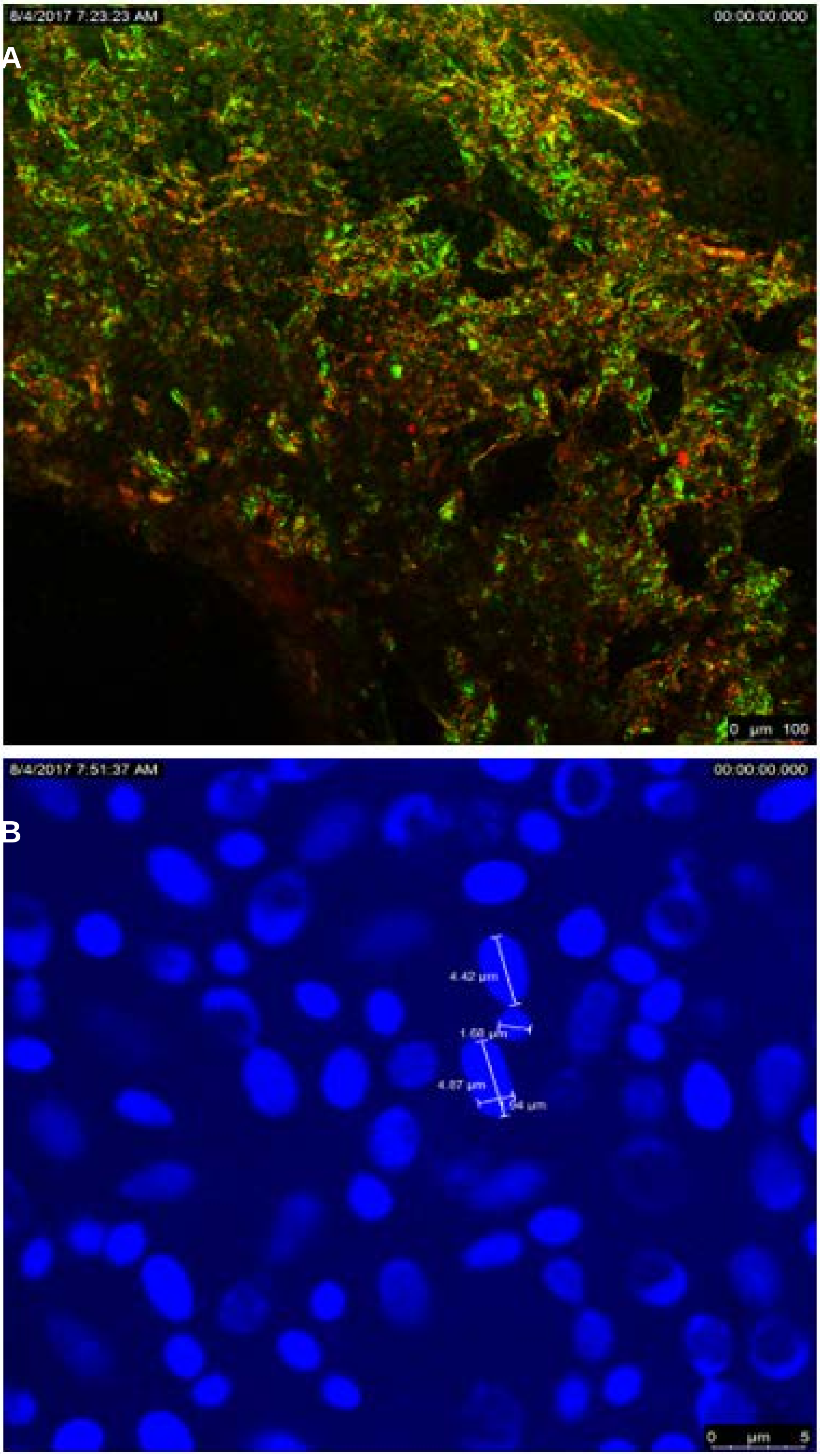

Fig. S2. Confocal microscopical view of *U. virens* interaction with the host using WGA and PI fluorescent stains (A) and the chlamydospores on germination to form ascospores and secondary conidial cells (B). bar; A = 100 µm, B = 5 µm.

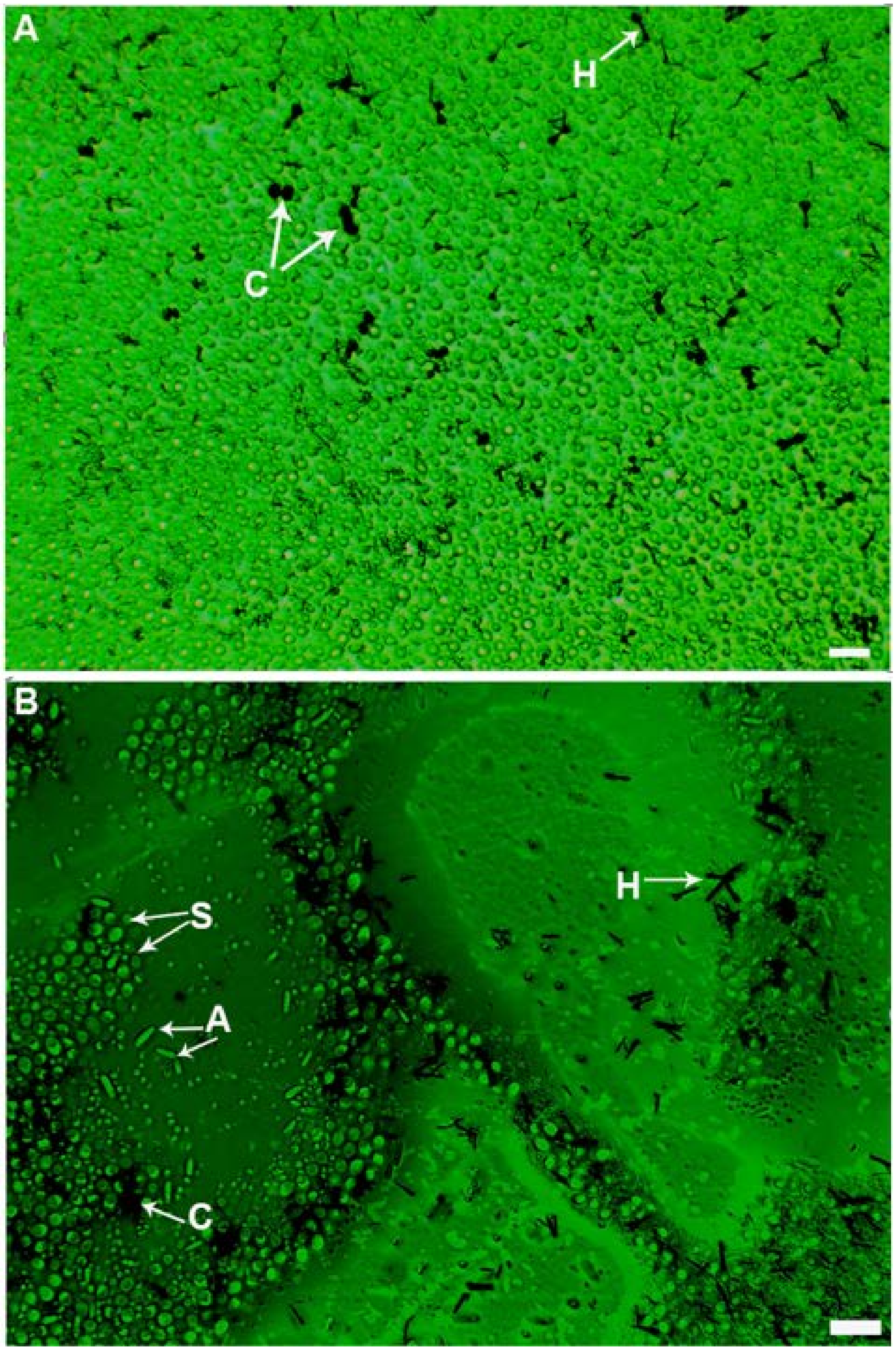

Fig. S3. Light microscopic view of *U. virens* chlamydospores (C) on germination to form Hyphae (H) ascospores (A) and secondary conidial cells (S) cells. bar; = 20 µm.

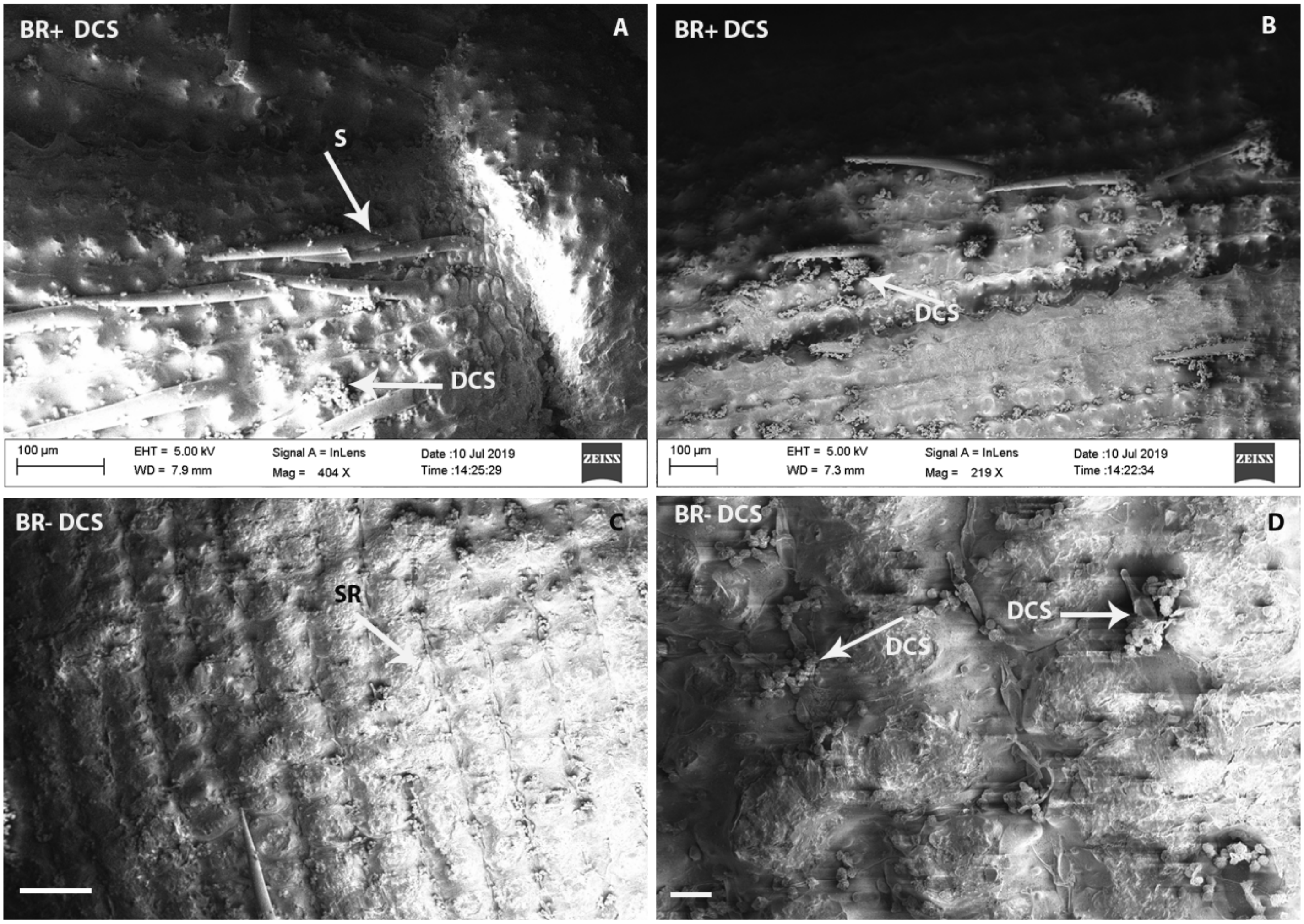

Fig. S4 SEM micrographs depicting the surface of rice grains scrapped with a surgical blade to reveal the interactions with dry chlamydospores (DCS/ C). Resistant Black rice is BR+ with spikes (S), and resistant Black rice BR- without spikes. The scale bar for **A-C** = 100 µm and **D** = 20 µm.

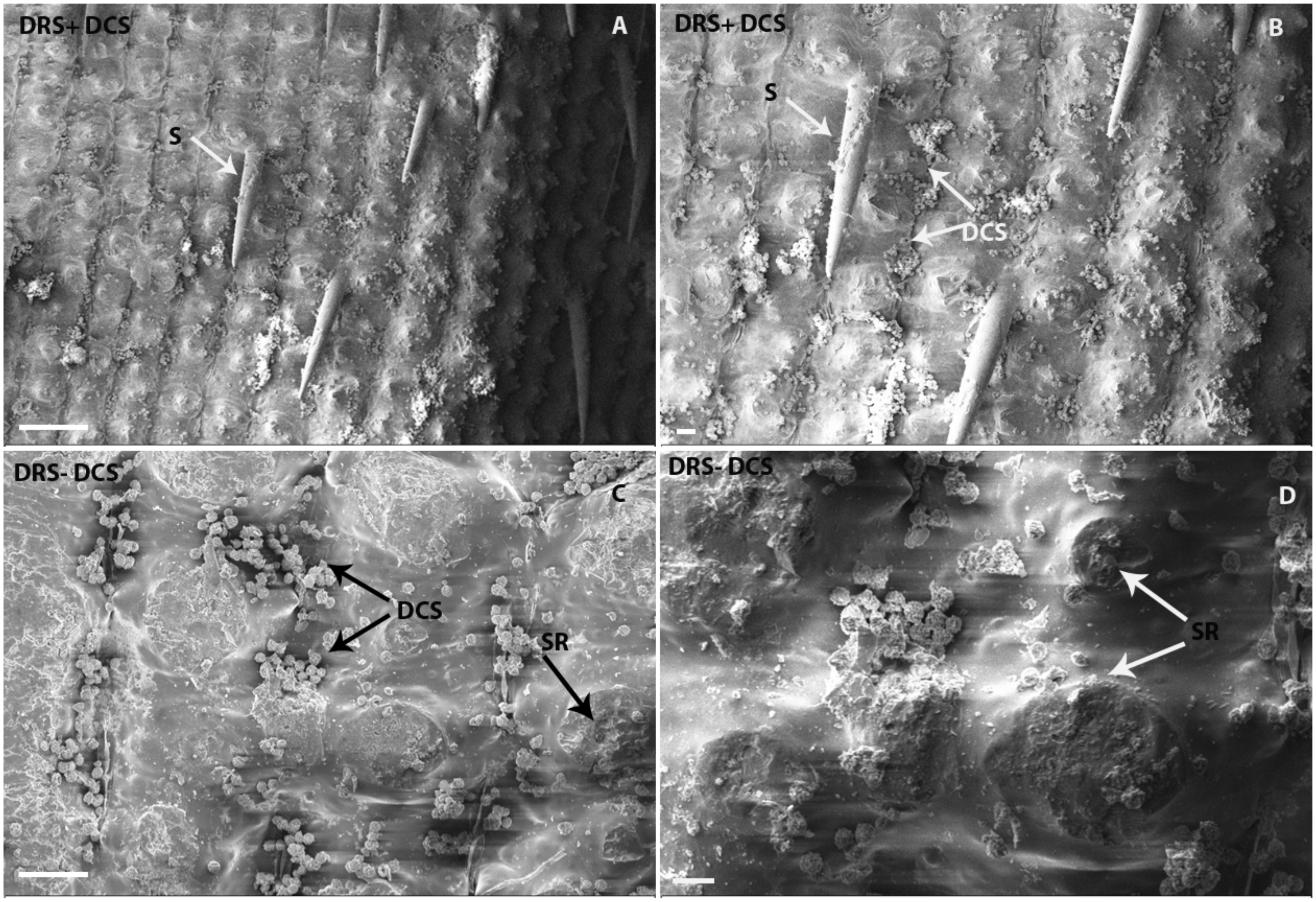

Fig. S5. SEM micrographs for susceptible rice grain variety Dharam. (**A-B**) Spikes present. (**C-D**) Spikes removed (SR), revealing the binding of dry chlamudospores of *U. virens* (DCS). The scale bar for A = 100 µm, B = 20 µm, C = 30 µm, and D = 10 µm.

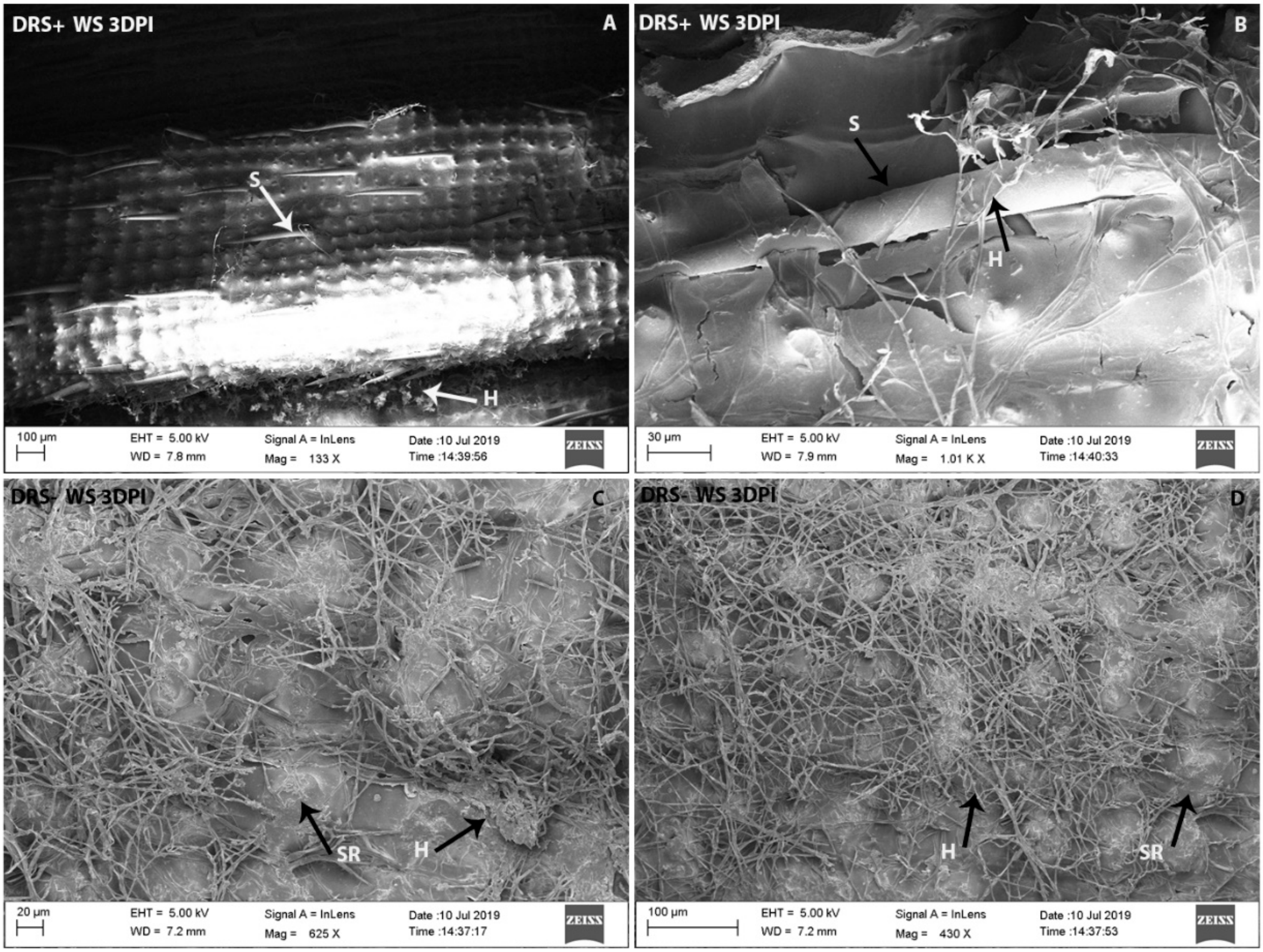

Fig. S6. SEM micrographs for susceptible rice grain variety Dharam (DRS). (**A-B**) Spikes present. (**C-D**) Spikes removed (SR), revealing the interaction with *U. virens* (DCS) 3 days post-inoculation. The scale bar for A = 100 µm, B = 20 µm, C = 30 µm, and D = 10 µm.

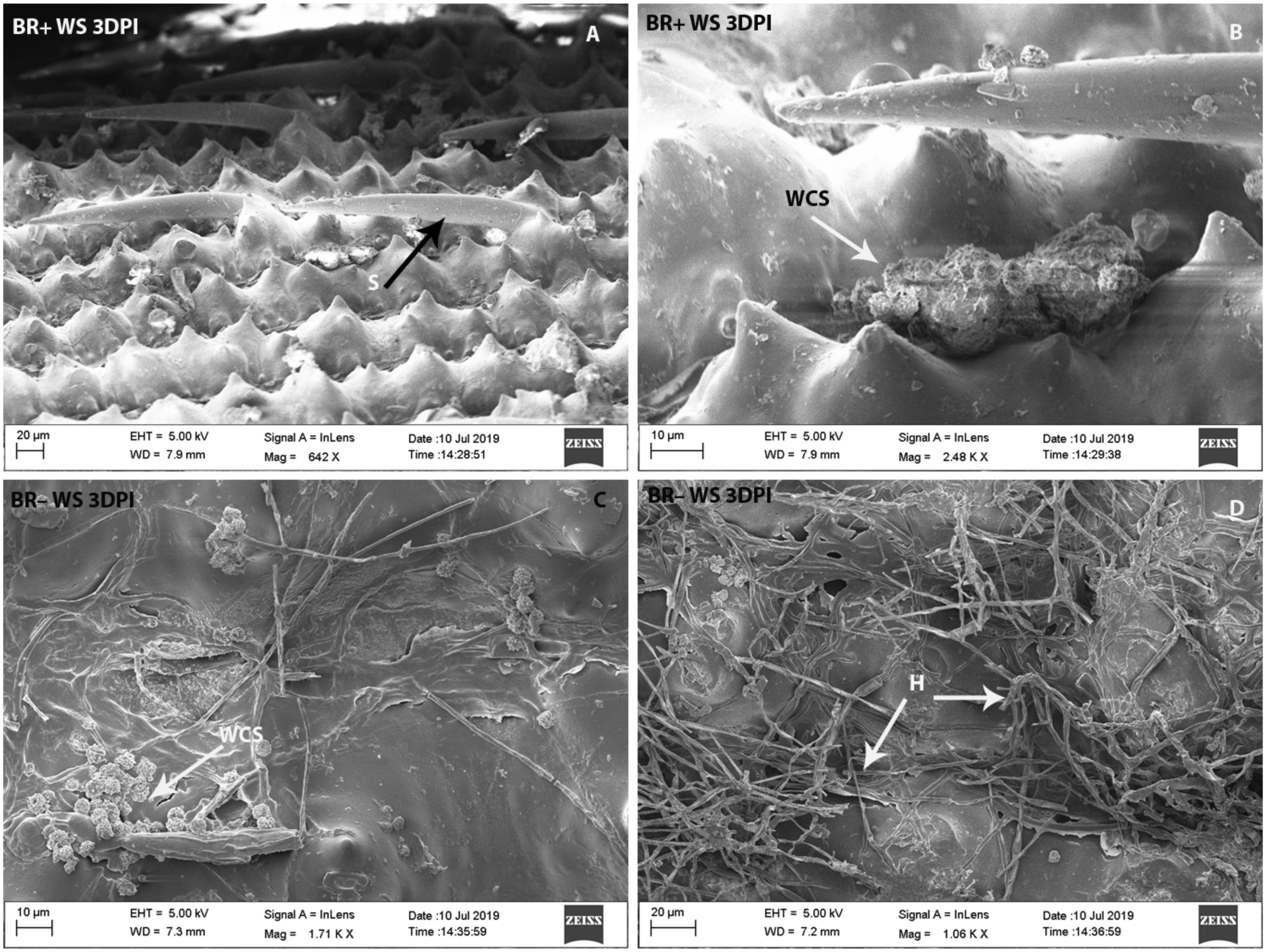

Fig. S7. SEM micrographs for resistant Black rice (BR+). (**A-B**) Spikes present. (**C-D**) Spikes removed (SR), revealing the interaction with chlamydospores (DCS) under wet conditions three days post-inoculation. The scale bar for A = 20 µm, B = 10 µm, C = 10 µm, and D = 20 µm. H is Hyphae.

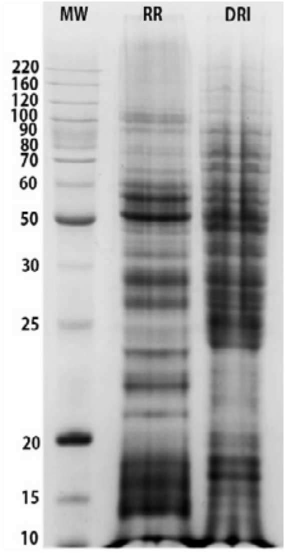

Fig. S8. SDS PAGE protein profile for uninfected Ranjit rice grains (RR) and infected Dharam rice (DRI) and the gel is calibrated with a molecular weight marker (MW).

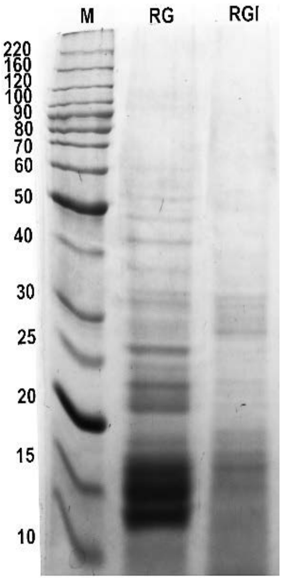

Fig. S9. SDS PAGE protein profile. Uninfected Thangjim (RG) rice grains and infected Thangjim (RGI) rice grains. The gel is calibrated with a molecular weight marker (MW).

Table S1. The frequency of the long spikes (LS) and small spikes (SS) on different grain varieties and the length and breadth in the center of the LS.

Grain Variety	LS per 200 μm^2	SS per 200 μm^2	Length of LS in µm	Diameter of LS in µm
BR	3 ± 1.41	19.2 ± 1.78	330.76 ± 1.24	23.62 ± 0.41
DS	1.6± 0.54	9 ± 0	278.36 ± 0.86	26.64 ± 0.19
RR	1.8 ± 1.30	10.8 ± 7.52	230.62 ± 1.14	24.49 ± 0.24
TS	1.2 ± 0.44	11.8 ± 3.03	253.66 ± 0.77	25.49 ± 0.43

Note: LS is long spikes, SS is short spikes, BR (Black rice), DS (Dharam rice), RR (Ranjit rice), and TS (Thangjing) are various rice varieties.

Table S2. Elemental analysis for rice varieties, quantification for short spikes (SS) and long spikes (LS).

Elements	Black rice		Ranjit Rice		Non-infected Dharam rice		Non-infected Thangjim rice	
	SS	LS	SS	LS	SS	LS	SS	LS
C %	17.2 ± 0.1	28.33 ± 0.11	13.59 ± 0.31	50.33 ± 0.15	30.39 ± 0.32	25.52 ± 0.25	25.89 ± 0.06	36.56 ± 0.25
O %	53.28 ± 0.20	48.33 ± 0.32	59.49 ± 0.34	21.72 ± 0.43	47.77 ± 0.27	43.73 ± 0.25	53.55 ± 0.01	55.81 ± 0.22
Si %	29.31 ± 0.17	23.39 ± 0.22	25.77 ± 0.12	27.83 ± 0.16	21.52 ± 0.41	30.55 ± 0.14	20.20 ± 0.16	7.77 ± 0.15

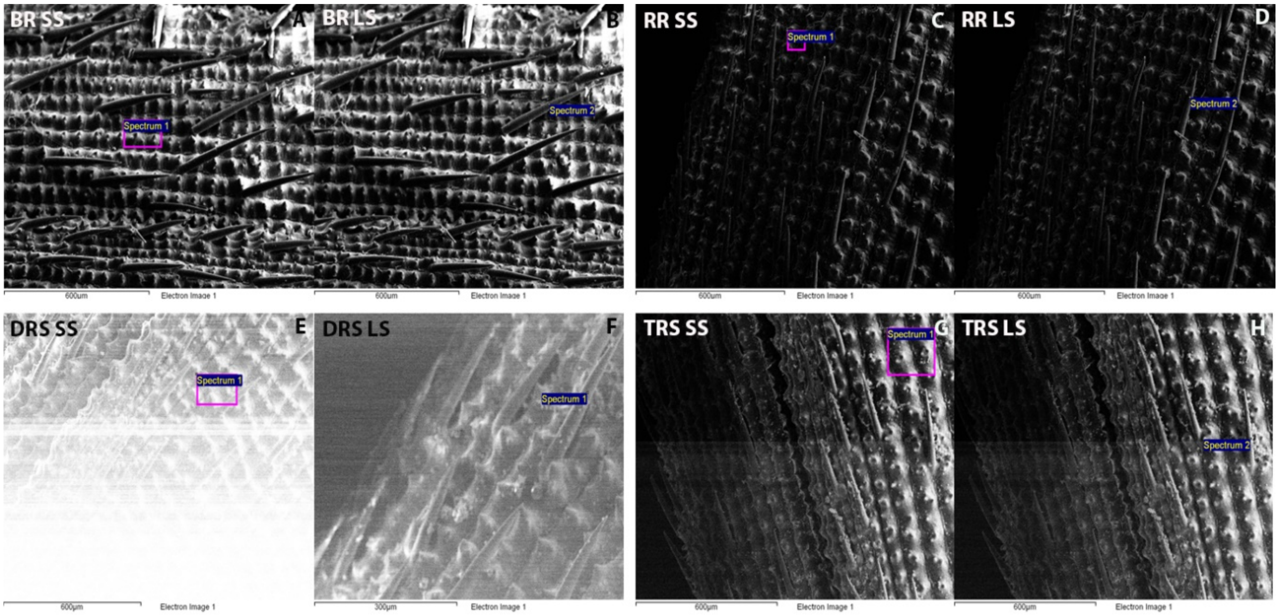

Table S3. The infection rate of two susceptible rice varieties, Dharam (DR) and Thangjim (TS) from Imphal State Manipur, India: Rice varieties such as Black rice (BR) and Ranjit Rice (RR) were resistant and did not have visible infected grains on the panicle.

Rice Variety	Grains in 1st Sampling	Grains in 2nd Sampling	Grains in 3rd Sampling	Infected grains	% Infection of all samplings
DR	201 ± 7.87	206.6 ± 9.78	209 ± 6.40	58.4 ± 116.61	9.66 ± 19.35
TS	139.6± 6.69	151.8 ± 19.13	152.2 ± 31.30	9.4 ± 3.50	2.14 ± 0.84
BR	265.6 ± 29.50	238 ± 21.67	236.4 ± 30.24	0	0
RR	204.8 ± 11.18	205.6 ± 19.66	210 ± 9.38	0	0

Table S4. Details of proteins detected by LC-MS/ MS from resistant and susceptible rice varieties and also from *U. virens* during false-smut formation.

**BAND 1**								
**Score**	**Mass**	**SC**	**NOS**	**NOSS**	**NOUS**	**NOSUS**	**emPAI**	**Description**
515	11362	0.5	4	4	4	4	4.1	Uncharacterized protein OS=*Oryza sativa* subsp. indica OX=39946 GN=OsI_27503 PE=4 SV=1
464	11767	0.56	4	4	4	4	3.8	OSIGBa0101P20.7 protein OS=*Oryza sativa* OX=4530 GN=OSIGBa0101P20.7 PE=4 SV=1
306	16859	0.38	3	3	3	3	1.28	Prolamin PPROL 14E OS=*Oryza sativa* subsp. indica OX=39946 PE=3 SV=1
257	10190	0.45	4	4	4	4	5.06	Uncharacterized protein OS= *Oryza sativa* subsp. indica OX=39946 GN=OsI_19553 PE=4 SV=1
191	24057	0.32	6	6	6	6	2.2	1-Cys peroxiredoxin A OS= *Oryza sativa* subsp. indica OX=39946 GN=OsI_27030 PE=2 SV=1
**BAND 2**								
**Score**	**Mass**	**SC**	**NOS**	**NOSS**	**NOUS**	**NOSUS**	**emPAI**	**Description**
76	23716	0.1	2	2	2	2	0.48	Uncharacterized protein OS= *Oryza sativa* subsp. indica OX=39946 GN=OsI_26825 PE=4 SV=1
67	24228	0.05	1	1	1	1	0.21	1-Cys peroxiredoxin A OS= *Oryza sativa* subsp. indica OX=39946 GN=OsI_27030 PE=2 SV=1
63	17838	0.16	2	2	2	2	0.68	Allergen RA5B OS= *Oryza sativa* subsp. japonica OX=39947 GN=Os07g0215500 PE=2 SV=1

60	15190	0.07	1	1	1	1	0.36	Oleosin OS= *Oryza sativa* OX=4530 GN=OSIGBa0101C23.13 PE=3 SV=1
55	10190	0.13	1	1	1	1	0.57	Uncharacterized protein OS= *Oryza sativa* subsp. indica OX=39946 GN=OsI_19553 PE=4 SV=1
54	16835	0.06	1	1	1	1	0.32	Nucleoside diphosphate kinase 1 OS= *Oryza sativa* subsp. indica OX=39946 GN=NDKR PE=1 SV=1
53	17542	0.1	1	1	1	1	0.3	Uncharacterized protein OS= *Oryza sativa* subsp. indica OX=39946 GN=OsI_25372 PE=4 SV=1
51	17829	0.06	1	1	1	1	0.3	Uncharacterized protein OS= *Oryza sativa* subsp. indica OX=39946 GN=OsI_25366 PE=4 SV=1
48	19471	0.05	1	1	1	1	0.27	Uncharacterized protein OS= *Oryza sativa* subsp. indica OX=39946 GN=OsI_28556 PE=4 SV=1
48	16496	0.05	1	1	1	1	0.32	Limit dextrinase inhibitor OS=Oryza sativa subsp. indica OX=39946 GN=OsI_25389 PE=4 SV=1
41	16458	0.06	1	1	1	1	0.33	Uncharacterized protein OS= *Oryza sativa* subsp. indica OX=39946 GN=OsI_36717 PE=4 SV=1
41	21497	0.04	1	1	1	1	0.24	Uncharacterized protein OS= *Oryza sativa* subsp. indica OX=39946 GN=OsI_20488 PE=4 SV=1
38	29253	0.04	1	1	1	1	0.17	Bowman-Birk type bran trypsin inhibitor OS= *Oryza sativa* subsp. japonica OX=39947 GN=RBBI3.3 PE=1 SV=2
**BAND 3**								
**Score**	**Mass**	**SC**	**NOS**	**NOSS**	**NOUS**	**NOSUS**	**emPAI**	**Description**

448	17211	0.33	5	5	5	5	2.86	Oleosin 18 kDa OS= *Oryza sativa* subsp. indica OX=39946 GN=OLE18 PE=3 SV=1
200	18067	0.22	3	3	3	3	1.16	Os03g0854400 protein OS= *Oryza sativa* subsp. japonica OX=39947 GN=Os03g0854400 PE=4 SV=1
180	18682	0.28	3	3	1	1	1.11	Cold shock domain protein 2 OS= *Oryza sativa* subsp. japonica OX=39947 GN=P0582D05.112 PE=2 SV=1
188	16264	0.26	3	3	3	3	1.35	Peptidylprolyl isomerase OS= *Oryza sativa* subsp. indica OX=39946 GN=OsI_31925 PE=4 SV=1
176	16769	0.47	6	6	6	6	4.28	Nucleoside diphosphate kinase OS= *Oryza sativa* subsp. indica OX=39946 GN=OsI_34670 PE=2 SV=1
**BAND 4**								
**Score**	**Mass**	**SC**	**NOS**	**NOSS**	**NOUS**	**NOSUS**	**emPAI**	**Description**
1330	15242	0.4	3	3	2	2	1.5	Superoxide dismutase [Cu-Zn] 1 OS= *Oryza sativa* subsp. japonica OX=39947 GN=SODCC1 PE=1 SV=1
918	15071	0.4	3	3	2	2	1.51	Superoxide dismutase [Cu-Zn] 2 OS= *Oryza sativa* subsp. japonica OX=39947 GN=SODCC2 PE=1 SV=2
1087	18349	0.3	5	5	5	5	2.56	Peptidyl-prolyl cis-trans isomerase OS= *Oryza sativa* subsp. indica OX=39946 GN=OsI_05635 PE=2 SV=1
645	18440	0.44	5	5	3	3	2.53	Uncharacterized protein OS= *Oryza sativa* subsp. indica OX=39946 GN=OsI_27689 PE=4 SV=1

522	19300	0.22	4	4	4	4	1.62	Uncharacterized protein OS= *Oryza sativa* subsp. indica OX=39946 GN=OsI_28556 PE=4 SV=1
469	16929	0.55	6	6	6	6	4.16	Uncharacterized protein OS= *Oryza sativa* subsp. indica OX=39946 GN=OsI_00285 PE=3 SV=1
343	17899	0.32	4	4	1	1	1.83	17.9 kDa class I heat shock protein OS= *Oryza sativa* subsp. japonica OX=39947 GN=HSP17.9A PE=1 SV=1
269	17647	0.31	4	4	2	2	1.87	17.7 kDa class I heat shock protein OS= *Oryza sativa* subsp. japonica OX=39947 GN=HSP17.7 PE=2 SV=1
118	18071	0.3	4	4	2	2	1.8	18.1 kDa class I heat shock protein OS= *Oryza sativa* subsp. japonica OX=39947 GN=HSP18.1 PE=2 SV=1
116	17634	0.24	3	3	1	1	1.2	Heat shock protein Hsp20 OS= *Oryza sativa* subsp. japonica OX=39947 PE=2 SV=1
421	17737	0.41	5	5	4	4	2.7	OSJNba0093F12.16 protein OS= *Oryza sativa* subsp. japonica OX=39947 GN=Os04g0598200 PE=3 SV=1
163	19759	0.13	2	2	1	1	0.6	60S ribosomal protein L12 OS=*Ustilaginoidea virens* OX=1159556 GN=UV8b_7599 PE=3 SV=1
390	16229	0.39	4	4	4	4	2.15	Os01g0225600 protein OS=*Oryza sativa* subsp. japonica OX=39947 GN=Os01g0225600 PE=4 SV=1
339	15273	0.45	4	4	3	3	2.36	Uncharacterized protein OS=*Oryza sativa* subsp. indica OX=39946 GN=OsI_18149 PE=3 SV=1

161	15010	0.32	3	3	2	2	1.53	Cytochrome b5 OS=*Oryza sativa* subsp. indica OX=39946 GN=OsI_05385 PE=2 SV=1
333	23449	0.34	7	7	6	6	3.04	Peptidyl-prolyl cis-trans isomerase OS=*Oryza sativa* subsp. indica OX=39946 GN=OsI_24414 PE=3 SV=1
720	20519	0.43	5	5	5	5	2.11	Superoxide dismutase [Cu-Zn] OS=*Oryza sativa* subsp. indica OX=39946 GN=OsI_30285 PE=3 SV=1
276	16250	0.47	6	6	6	6	4.58	Alba1 OS=*Oryza sativa* subsp. indica OX=39946 GN=Alba1 PE=2 SV=1
204	20502	0.23	4	4	4	4	1.48	Late embryogenesis abundant protein 19 OS=*Oryza sativa* subsp. japonica OX=39947 GN=LEA19 PE=2 SV=1
183	18826	0.28	4	4	4	4	1.68	Uncharacterized protein OS=*Oryza sativa* subsp. indica OX=39946 GN=OsI_33745 PE=4 SV=1
182	15154	0.48	4	4	4	4	2.39	Os08g0327700 protein OS=*Oryza sativa* subsp. japonica OX=39947 GN=Os08g0327700 PE=2 SV=1
179	17760	0.35	5	5	2	2	2.7	Os08g0117200 protein (Fragment) OS=*Oryza sativa* subsp. japonica OX=39947 GN=Os08g0117200 PE=3 SV=1
124	17135	0.41	6	6	3	3	4.11	40S ribosomal protein S13-2 OS=*Oryza sativa* subsp. japonica OX=39947 GN=Os08g0117300 PE=1 SV=1
177	19645	0.13	1	1	1	1	0.27	Uncharacterized protein OS=*Oryza sativa* subsp. indica OX=39946 GN=OsI_13912 PE=3 SV=1
176	21879	0.3	5	5	5	5	1.91	Os12g0626500 protein OS=*Oryza sativa* subsp. japonica OX=39947 GN=Os12g0626500 PE=4 SV=1

176	18596	0.42	5	5	5	5	2.48	Cyanate hydratase OS=*Oryza sativa* subsp. indica OX=39946 GN=CYN PE=3 SV=1
**BAND 5**								
**Score**	**Mass**	**SC**	**NOS**	**NOSS**	**NOUS**	**NOSUS**	**emPAI**	**Description**
893	18234	0.59	6	6	6	6	3.63	Os05g0468800 protein OS=*Oryza sativa* subsp. japonica OX=39947 GN=Os05g0468800 PE=4 SV=1
595	18359	0.61	7	7	7	7	4.92	Mitochondrial import inner membrane translocase subunit Tim17/Tim22/Tim23 family protein, putative, expressed OS=*Oryza sativa* subsp. japonica OX=39947 GN=Os03g0305600 PE=2 SV=1
591	21404	0.34	5	5	5	5	1.97	OSIGBa0115K01-H0319F09.5 protein OS=*Oryza sativa* OX=4530 GN=OSIGBa0115K01-H0319F09.5 PE=4 SV=1
338	27144	0.56	8	8	8	8	2.96	Uncharacterized protein OS=*Oryza sativa* subsp. indica OX=39946 GN=OsI_10103 PE=4 SV=1
334	18906	0.34	3	3	3	3	1.1	Putative stress-related protein OS=*Oryza sativa* subsp. japonica OX=39947 GN=OSJNBa0047E24.27 PE=4 SV=1
315	21849	0.42	6	6	6	6	2.6	Uncharacterized protein OS=*Oryza sativa* subsp. indica OX=39946 GN=OsI_39200 PE=4 SV=1
267	24470	0.42	6	6	6	6	2.15	Uncharacterized protein OS=*Oryza sativa* subsp. indica OX=39946 GN=OsI_01378 PE=4 SV=1
262	22119	0.14	2	2	2	2	0.53	Group 3 late embryogenesis abundant protein OS=*Oryza sativa* subsp. indica OX=39946 GN=Wsi18 PE=2 SV=1

261	27861	0.43	6	6	6	6	1.74	Uncharacterized protein OS=*Oryza sativa* subsp. indica OX=39946 GN=OsI_22847 PE=4 SV=1
226	19300	0.22	4	4	4	4	1.62	Uncharacterized protein OS=*Oryza sativa* subsp. indica OX=39946 GN=OsI_28556 PE=4 SV=1
207	19713	0.44	7	7	7	7	4.21	ATP synthase subunit d, mitochondrial OS=*Oryza sativa* subsp. japonica OX=39947 GN=Os08g0478200 PE=2 SV=1
**BAND 6**								
**Score**	**Mass**	**SC**	**NOS**	**NOSS**	**NOUS**	**NOSUS**	**emPAI**	**Description**
77	23716	0.05	1	1	1	1	0.22	Uncharacterized protein OS=*Oryza sativa* subsp. indica OX=39946 GN=OsI_26825 PE=4 SV=1
55	24228	0.05	1	1	1	1	0.21	1-Cys peroxiredoxin A OS=*Oryza sativa* subsp. indica OX=39946 GN=OsI_27030 PE=2 SV=1
52	16835	0.06	1	1	1	1	0.32	Nucleoside diphosphate kinase 1 OS=*Oryza sativa* subsp. indica OX=39946 GN=NDKR PE=1 SV=1
50	17542	0.1	1	1	1	1	0.3	Uncharacterized protein OS=*Oryza sativa* subsp. indica OX=39946 GN=OsI_25372 PE=4 SV=1
50	59270	0.02	1	1	1	1	0.08	Uncharacterized protein OS=*Oryza sativa* subsp. indica OX=39946 GN=OsI_12896 PE=4 SV=1
46	57892	0.01	1	1	1	1	0.08	Uncharacterized protein OS=*Oryza sativa* subsp. indica OX=39946 GN=OsI_27675 PE=4 SV=1
**BAND 7**								

Score	Mass	SC	NOS	NOSS	NOUS	NOSUS	emPAI	Description
2417	40127	0.45	14	14	14	14	4.76	Subtilisin-like protease pr1a OS=*Ustilaginoidea virens* OX=1159556 GN=UV8b_1567 PE=3 SV=1
859	41218	0.35	9	9	9	9	2.12	GPI-anchored cell wall beta-1,3-endoglucanase EglC OS= *Ustilaginoidea virens* OX=1159556 GN=UV8b_6459 PE=4 SV=1
411	39645	0.23	5	5	5	5	1.29	Subtilisin-like protease pr1a OS=*Ustilaginoidea virens* OX=1159556 GN=UV8b_44 PE=3 SV=1
268	32258	0.33	7	7	7	7	1.76	Os04g0404400 protein (Fragment) OS=*Oryza sativa* subsp. japonica OX=39947 GN=Os04g0404400 PE=4 SV=1
208	33781	0.29	9	9	9	9	2.49	Putative nuclease S1 OS=*Ustilaginoidea virens* OX=1159556 GN=UV8b_1810 PE=4 SV=1
176	37021	0.18	5	5	5	5	1.14	Transaldolase OS=*Ustilaginoidea virens* OX=1159556 GN=UVI_02016190 PE=4 SV=1
148	29978	0.25	5	5	5	5	1.55	TH14-3-3 like protein OS=*Ustilaginoidea virens* OX=1159556 GN=UV8b_5776 PE=3 SV=1
145	64232	0.11	5	5	5	5	0.44	2-isopropylmalate synthase B, putative, expressed OS=*Oryza sativa* subsp. japonica OX=39947 GN=LOC_Os12g04440 PE=3 SV=1
143	34727	0.23	5	5	5	5	1.25	Putative palmitoyl-protein thioesterase OS=*Ustilaginoidea virens* OX=1159556 GN=UV8b_2012 PE=4 SV=1
132	40946	0.09	2	2	2	2	0.41	ECM33-like protein OS=*Ustilaginoidea virens* OX=1159556 GN=UV8b_3724 PE=4 SV=1

116	36714	0.16	3	3	3	3	0.47	Adenosine kinase OS=*Ustilaginoidea virens* OX=1159556 GN=UV8b_1703 PE=4 SV=1
**BAND 8**								
**Score**	**Mass**	**SC**	**NOS**	**NOSS**	**NOUS**	**NOSUS**	**emPAI**	**Description**
427	16654	0.4	5	5	5	5	3.04	Cnd01770-like protein OS=*Ustilaginoidea virens* OX=1159556 GN=UV8b_1318 PE=4 SV=1
196	15026	0.24	2	2	2	2	0.86	Ester cyclase OS=*Ustilaginoidea virens* OX=1159556 GN=UV8b_4649 PE=4 SV=1
158	16094	0.19	2	2	2	2	0.78	Superoxide dismutase [Cu-Zn] OS=*Ustilaginoidea virens* OX=1159556 GN=UV8b_988 PE=3 SV=1
157	19494	0.3	4	4	4	4	1.59	Uncharacterized protein OS=*Ustilaginoidea virens* non segmented virus 1 OX=1520125 PE=4 SV=1
156	17153	0.22	3	3	3	3	1.25	Small secreted protein OS=*Ustilaginoidea virens* OX=1159556 GN=UV8b_1554 PE=4 SV=1
132	17877	0.22	2	2	2	2	0.68	Bys1 family protein OS=*Ustilaginoidea virens* OX=1159556 GN=UV8b_2621 PE=4 SV=1
117	15242	0.32	2	2	1	1	0.84	Superoxide dismutase [Cu-Zn] 1 OS=*Oryza sativa* subsp. indica OX=39946 GN=SODCC1 PE=2 SV=2
128	16769	0.35	4	4	4	4	2.03	Nucleoside diphosphate kinase OS=*Oryza sativa* subsp. indica OX=39946 GN=OsI_34670 PE=2 SV=1

83	16835	0.22	3	3	3	3	1.28	Nucleoside diphosphate kinase 1 OS=*Oryza sativa* subsp. indica OX=39946 GN=NDKR PE=1 SV=1
125	21916	0.25	4	4	4	4	1.35	Uncharacterized protein OS=*Oryza sativa* subsp. indica OX=39946 GN=OsI_12872 PE=4 SV=1
119	39360	0.18	3	3	3	3	0.43	Subtilisin-like protease pr1a OS=*Ustilaginoidea virens* OX=1159556 GN=UV8b_44 PE=3 SV=1
118	61659	0.11	5	5	5	5	0.46	Peroxidase OS=*Ustilaginoidea virens* OX=1159556 GN=UV8b_5622 PE=3 SV=1
111	16035	0.31	4	4	4	4	2.2	Small secreted protein OS=*Ustilaginoidea virens* OX=1159556 GN=UV8b_6932 PE=4 SV=1
105	17946	0.19	2	2	2	2	0.68	DNase1 protein OS=*Ustilaginoidea virens* OX=1159556 GN=UV8b_187 PE=4 SV=1

Note: SC = Sequence coverage, NOS = Number of sequences, NOSS = Number of significant sequences, NOUS = Number of unique sequences, NOSUS = Number of significant unique sequences, emPAI = Exponentially modified protein abundance index
